# Molecular characterization of canine parvovirus variants (CPV-2a, CPV-2b, and CPV-2c) based on the *VP2* gene in affected domestic dogs in Ecuador

**DOI:** 10.14202/vetworld.2018.480-487

**Published:** 2018-04-16

**Authors:** David De la Torre, Eulalia Mafla, Byron Puga, Linda Erazo, Claudete Astolfi-Ferreira, Antonio Piantino Ferreira

**Affiliations:** 1 Department of Pathology, School of Veterinary Medicine, University of São Paulo, Av. Prof. Orlando Marques de Paiva, 87, CEP 05508-270, São Paulo, Brazil; 2 Department of Pathology, School of Veterinary Medicine and Animal Science, Central University of Ecuador, EC170521, Quito, Ecuador

**Keywords:** canine parvovirus, canine parvovirus-2, Ecuador, molecular characterization, variants

## Abstract

**Aim:**

The objective of this study was to determine the presence of the variants of canine parvovirus (CPV)-2 in the city of Quito, Ecuador, due to the high domestic and street-type canine population, and to identify possible mutations at a genetic level that could be causing structural changes in the virus with a consequent influence on the immune response of the hosts.

**Materials and Methods:**

Thirty-five stool samples from different puppies with characteristic signs of the disease and positives for CPV through immunochromatography kits were collected from different veterinarian clinics of the city. Polymerase chain reaction and DNA sequencing were used to determine the mutations in residue 426 of the *VP2* gene, which determines the variants of CPV-2; in addition, four samples were chosen for complete sequencing of the *VP2* gene to identify all possible mutations in the circulating strains in this region of the country.

**Results:**

The results revealed the presence of the three variants of CPV-2 with a prevalence of 57.1% (20/35) for CPV-2a, 8.5% (3/35) for CPV-2b, and 34.3% (12/35) for CPV-2c. In addition, complete sequencing of the *VP2* gene showed amino acid substitutions in residues 87, 101, 139, 219, 297, 300, 305, 322, 324, 375, 386, 426, 440, and 514 of the three Ecuadorian variants when compared with the original CPV-2 sequence.

**Conclusion:**

This study describes the detection of CPV variants in the city of Quito, Ecuador. Variants of CPV-2 (2a, 2b, and 2c) have been reported in South America, and there are cases in Ecuador where CVP-2 is affecting even vaccinated puppies.

## Introduction

Canine parvovirus (CPV) is a common etiological agent that causes severe gastroenteritis in young dogs, especially unvaccinated puppies or those with poor maternal protection through passive immunity [1,2]. Parvoviruses replicate mainly in intestinal crypts and the lymphoid organs but may reach any organ in susceptible animals. The most characteristic signs of this illness are diarrhea, emesis, anorexia, depression, pyrexia, or hypothermia [3,4]. CPV belongs to the genus Protoparvovirus within the family Parvoviridae, which includes the species *Carnivore protoparvovirus* 1 together with the feline panleukopenia virus (FPV), mink enteritis virus, and raccoon parvovirus [5]. The viral particle shows a spherical capsid and non-enveloped structure, with a diameter of 25 nm. The genome consists of a linear and single-stranded DNA molecule that is 5.2 kb in length, with two open reading frames (ORF) [2,6]. The first ORF encodes two non-structural proteins (NS1 and NS2) and the second ORF encodes two structural proteins (VP1 and VP2) that assemble the capsid with 54 units of VP1 and 6 units of VP2 [7]. CPV is a variant of the FPV with the new nomenclature of canine parvovirus type 2 (CPV-2) and differs genetically and antigenically from the canine minute virus, designed as CPV-1, which causes neonatal death in dogs [5]. CPV-2 became widespread since 1978, and using monoclonal antibodies, CPV-2a and CPV-2b variants were reported around the world since 1980, especially in the United States, Belgium, France, Australia, and Japan [8,9].

In 2000, a third variant, designated CPV-2c, was reported in Europe using molecular methods [10]. There were several mutations in the genome between the three variants and the original CVP-2, but one, in particular, has been described in codon 426 within the VP2 segment in ORF2, which consisted of the substitutions Asn (CPV-2 and CPV-2a), Asp (CPV-2b), and Glu (CPV-2c) [9-11]. All three variants (2a, 2b, and 2c) have been reported in South America since 2007, affecting young dogs from all breeds [12-16]. Immunochromatography tests, polymerase chain reaction (PCR), nucleotide sequencing, and virus isolation in Madin-Darby canine kidney cells are commonly used to detect and characterize CPVs [17-21].

The aim of this work was to detect and establish the molecular characterization of CPV affecting domestic dogs in Ecuador, based on the substitution of residue 426 in the *VP2* gene sequence, and comparing all amino acid substitutions in complete *VP2* gene with previous sequences reported in Ecuador and some selected countries around the world.

## Materials and Methods

### Ethical approval

Samples from animals were collected in a non-invasive manner and authorized for research use by the owners. All procedures were conducted according to the regulations of the Ethical Commission for Animal Use of the School of Veterinary Medicine, University of São Paulo.

### Samples

The samples were collected from different clinics located in Quito city during the months of May, June, and July in 2017 by veterinarians specialized in medicine for dogs and cats. A total of 35 fecal samples and four commercial vaccines for positive controls (C1-C4) (Canigen MHA_2_Puppy, HIPRADOG 7, Nobivac PUPPY DP, and Vanguard^®^ Plus CPV/CV) were fixed in FTA cards (GE Healthcare Company, Little Chalfont, Buckinghamshire, UK), accompanied by a basic record for each animal, filled in with information regarding age, breed, geographical zone, gender, and vaccination status. All animals reported clinical signs associated with parvovirus infections such as gastroenteritis, anorexia, and vomiting. Parvovirus was confirmed by detection with the SNAP^®^ Parvo Test (Idexx Laboratories, Westbrook, Maine, USA) before the fixation of feces in the FTA cards. All the material was stored in individual plastic bags and sent for molecular analysis to the Department of Pathology, School of Veterinary Medicine at the University of São Paulo, Brazil. All data from each sample are available in Table-1.

**Table-1:** Data obtained from each positive sample for CPV.

Clinical case number	Age (weeks)	Breed	Sex	Vaccinated	Variant CPV	Accession number
1	8	French poodle	Female	Yes	2a	MG264075
2	10	Mixed	Male	No	2a	MG264044
3	12	Mixed	Male	No	2c	MG264045
4	20	Mixed	Male	Yes	2a	MG264076
5	8	Miniature Pinscher	Female	No	2c	MG264046
6	28	Mixed	Female	No	2c	MG264047
7	8	Siberian Husky	Female	No	2b	MG264048
8	24	American bulldog	Male	Yes	2c	MG264077
9	16	Mixed	Female	No	2a	MG264049
10	8	Chihuahua	Male	Yes	2a	MG264050
11	8	Beagle	Male	No	2a	MG264051
12	10	Golden retriever	Male	NR	2a	MG264052
13	12	Mixed	Male	No	2c	MG264053
14	14	Mixed	Female	NR	2a	MG264054
15	28	Beagle	Female	NR	2a	MG264055
16	12	Mixed	Female	No	2a	MG264056
17	16	French poodle	Female	No	2b	MG264057
18	12	Mixed	Female	No	2a	MG264058
19	8	Siberian Husky	Female	Yes	2a	MG264059
20	12	Schnauzer	Male	No	2a	MG264060
21	12	Miniature Pinscher	Female	No	2c	MG264061
22	NR	NR	Male	No	2c	MG264062
23	24	Mixed	Male	No	2c	MG264063
24	20	Mixed	Male	No	2b	MG264078
25	12	Mixed	Female	NR	2a	MG264064
26	8	German shepherd	Male	NR	2a	MG264065
27	NR	Mixed	Male	No	2c	MG264066
28	24	American pit bull	Female	No	2c	MG264067
29	8	Mixed	Male	No	2a	MG264068
30	16	Mixed	Female	No	2a	MG264069
31	36	Mixed	Male	No	2a	MG264070
32	8	Mixed	Female	No	2c	MG264071
33	16	Mixed	Male	No	2a	MG264072
34	8	Mixed	Female	No	2a	MG264073
35	28	Akita	Female	Yes	2c	MG264074

CPV: Canine parvovirus, NR: Not reported

### DNA extraction

The circle inside the FTA card was cut with sterile scissors and suspended in 500 µl of phosphate-buffered solution, 0.1 M, pH 7.4. The material was macerated using the TissueLyser LT Bead Mill (Qiagen, Hilden, Germany) instrument for 5 min. The suspension was centrifuged for 30 min at 12,000× *g*, and 200 µl of supernatant was collected for DNA extraction using the phenol/chloroform method [22]. Extracted DNA was stored at −20°C for further analysis.

### PCR and DNA sequencing

**Figure-1:**
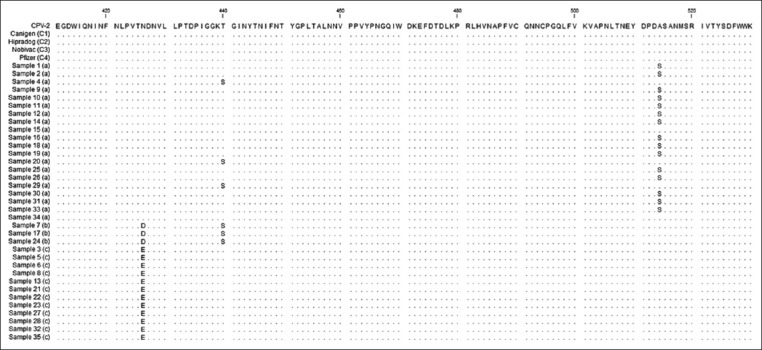
Alignment performed with the Clustal W method, using protein sequences of 35 samples, four positive controls, and the reference sequence of canine parvovirus variants type 2 from GenBank.

Two sets of primers were designed with the Primer3Plus free software [23] based on the viral genome that encodes the *VP2* protein. The first pair of primers was designed to amplify a region of *VP2* gene that involves residue 426, which determines the classification of the three variants of the CPV-2 (VP2-F 5’-AGCAGATGGTGATCCAAGAT-3’, and VP2-R 5’-TGGATTCCAAGTATGAGAGG-3’) and was used for amplification of all 35 samples and 4 positive controls. The PCR reactions were carried out with a mix that contained 2.5 µl of extracted DNA, 1× PCR Buffer - Mg, 1.25 mM of each deoxynucleotide triphosphate, 0.5 µM of each primer (VP2-F and VP2-R), 1 U of Platinum Taq DNA Polymerase (Invitrogen, Carlsbad, CA, USA), 1.5 mM of MgCl_2_, and enough ultrapure DNase-free distilled water to reach 25 µl of the mixture. The following temperature conditions were used for the PCR reactions: A cycle of 94°C for 4 min, 30 cycles at 94°C for 30 s, 56°C for 45 s, and 72°C for 1 min, followed by a final extension at 72°C for 10 min. The second pair of primers was designed to cover the complete *VP2* gene (cVP2-F 5’-GGTGCAGGACAAGTAAAAAGAG-3’ and cVP2-R 5’-ACCCACACCATAACAACATACA-3’) and was used in samples 1, 4, 8, and 24 and the Nobivac vaccine (C3). Sample 1 was chosen randomly from all samples with the mutation Ser514Ala (Figure-1), sample 4 was chosen randomly from CPV-2a samples affecting vaccinated dogs and without the mutation Ser514Ala (Figure-1), sample 8 was chosen randomly from CPV-2c samples affecting vaccinated dogs (Table-1), and sample 24 was chosen randomly from CPV-2b samples (Table-1). The following temperature conditions were used for the PCR reactions: A cycle of 95°C for 4 min, 35 cycles at 95°C for 45 s, 56°C for 2 min, and 72°C for 1 min, followed by a final extension at 72°C for 10 min. PCR products were analyzed by electrophoresis on 1% agarose gels with a 100 bp DNA ladder to estimate the fragment sizes of 529 bp for the first PCR product and 2191 bp for the second PCR product. The PCR products were purified using the GPX™ PCR DNA and Gel Band Purification kit (GE Healthcare, Piscataway, New Jersey, USA) following the manufacturer’s instructions. The purified products were submitted to the sequencing reaction in both directions, forward and reverse, using the BigDye^®^ Terminator Cycle Sequencing Kit v3.1 (Applied Biosystems by Life Technologies, Carlsbad, California, USA). Sequencing reactions were analyzed with an ABI 3730 DNA Analyzer (Applied Biosystems by Life Technologies).

### Phylogenetic and sequence analysis

**Figure-2:**
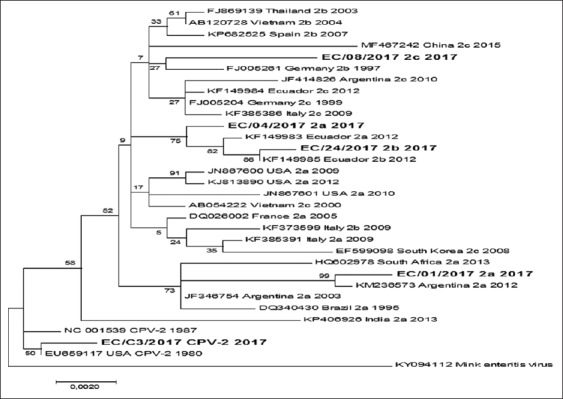
The evolutionary tree was inferred using the maximum-likelihood method. The percentage of trees in which the associated taxa clustered together is shown next to the branches. Initial tree for the heuristic search was obtained automatically by applying Neighbor-Join and BioNJ algorithms to a matrix of pairwise distances estimated using the maximum composite likelihood approach and then selecting the topology with superior log-likelihood value. A discrete gamma distribution was used to model evolutionary rate differences among sites.

Electropherograms obtained from the sequence readings were assembled and translated into protein sequences with CLC Main Work Bench 7.7.1 software. Nucleotide and amino acid (AA) sequences were aligned using the CLUSTAL W method available in Clustal X 2.0 software. *VP2* proteins of each VP2 AA complete sequences were structured in the SWISS-MODEL system [24], and residues were depicted using PyMOL software v.2.0.3. Phylogenetic analysis was inferred using the maximum likelihood statistical method integrated into MEGA 7.0.18 [25] with 1000 bootstrap replications [26]. Accession codes for sequences of positive controls are as follows: MG264041 (Canigen), MG264042 (Hipradog), MG264079 (Nobivac), and MG264043 (Pfizer). Complete VP2 sequences (1755 bp) were used to infer the phylogenetic tree and reference sequences were taken from the GenBank database which belonged to strains isolated from different countries around the world (Figure-2).

## Results

### Data analysis

Dog breeds used in this study were not specifically selected and depended on the availability of each veterinarian clinic that helped us. A number of 19/35 (54.3%) mixed dogs and 16/35 (45.7%) dogs corresponding to different breeds without a specific pattern were obtained. Regarding age, 10/35 (28.5%) were 8 weeks’ old, 2/35 (5.7%) were 10 weeks’ old, 7/35 (20%) were 12 weeks’ old, 5/35 (14.3%) were between 14 and 16 weeks’ old, and the remaining 9/35 (25.7%) were between 20 and 36 weeks’ old, with 2/35 final samples without information about the age of the dogs. Of the total, 17/35 (48.5%) animals were males and 18/35 (51.5%) were females. According to the information provided by the owner of each animal, 6/35 (17.1%) of them received an immunization with a commercial vaccine against parvoviruses, with the number of doses or any other report about the vaccination program of their pets not provided.

### PCR and DNA sequencing

**Figure-3:**
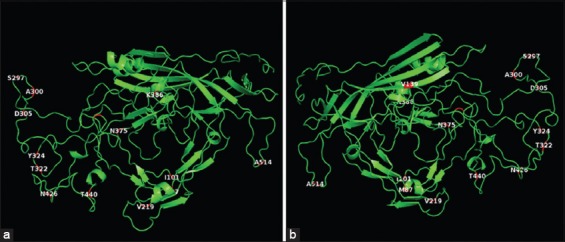
Ribbon diagrams of VP2 from C3 (canine parvovirus variants type 2), with the front (a) and rear (b) views, in which the 14 residues with mutations between our variants are displayed (red sections).

**Figure-4:**
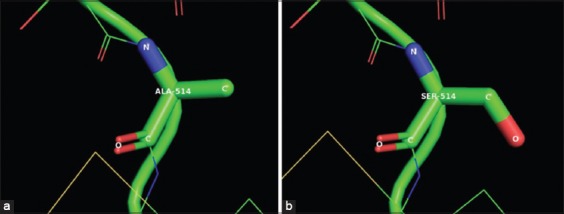
Comparison of residue 514 from VP2 in a zoomed-in view of the ribbon diagram, between the C3 (a) and sample 1 (b), showing the substitution from alanine (Ala) in C3 (canine parvovirus variants type 2 [CPV-2]) to serine (Ser) in sample 1 (CPV-2a).

All samples showed amplification of the target fragments in the first and second round PCRs (529 bp and 2191 bp, respectively) which were confirmed by agarose gel electrophoresis. The nucleotide sequences of the 35 samples and the 4 positive controls, obtained in the first round of PCRs, were translated into protein sequences, and they were aligned with a reference sequence (NP_955539) of CPV-2a obtained from GenBank. The size of the protein sequences obtained for each sample after the assembly and translation of the nucleotide sequences was approximately 168 aa, starting at residue 376 until residue 543 of the *VP2* gene. Based on the mutation located at residue 426 of CPV-2b (Asn426Asp) and CPV-2c (Asn426Glu), we found that 20/35 (57.1%) samples corresponded to CPV-2a, 12/35 (34.3%) to CPV-2c, and the remaining 3/35 (8.5%) to the CPV-2b variants. According to the aa alignment (Figure-1), 15/20 (75%) samples corresponding to the CPV-2a variant showed a mutation in residue 514 (Ala-Ser), which differed from the remaining 24 sequences, including CPV-2b, CPV-2c, and the positive controls (CPV-2). To search for additional mutations in the VP2 genome from the three variants found in this study, an analysis of the complete sequence of the *VP2* gene was performed, comparing four samples selected as described in the materials and methods against the VP2 sequence of the virus present in the Nobivac (C3) vaccine corresponding to the original CPV-2. In addition, to describe the mutation in residue 426, several mutations were found in the *VP2* protein of samples 1, 4, 8, and 24 when compared with C3 (Nobivac): Met87Leu, Ile101Thr, Val219Ile, Ala300Gly, Asp305Tyr, Asn375Asp, and Lys386Gln. Sample 1 also showed additional mutations corresponding to Ser297Asn, Tyr324Ile, and Ala514Ser; samples 4, 8, and 24 had a mutation in residue Ser297Ala; samples 4 and 24 showed a mutation in Thr440Ser; sample 28 showed a mutation in residue Thr322Ser; and sample 8 showed a mutation in residue Val139Ile. All mutations in the *VP2* gene in the four samples are described in Table-2. The residues with mutations were localized in a three-dimensional (3D) figure of the *VP2* protein generated by the SWISS-MODEL system and depicted with PyMOL software (Figure-3). The substitution of residue 514 from alanine in C3 to serine in sample 1 is illustrated in a zoomed-in view of the ribbon diagram (Figure-4).

**Table-2:** Residue mutations present in protein sequences of samples 1, 4, 8, and 24, compared with the C3 (Nobivac vaccine).

Canine samples	Residues													

	87	101	139	219	297	300	305	322	324	375	386	426	440	514
CPV-2 (C3)	Met	Ile	Val	Val	Ser	Ala	Asp	Thr	Tyr	Asn	Lys	Asn	Thr	Ala
CPV-2a (1)	Leu	Thr	Val	Ile	Asn	Gly	Tyr	Thr	Ile	Asp	Gln	Asn	Thr	Ser
CPV-2a (4)	Leu	Thr	Val	Ile	Ala	Gly	Tyr	Thr	Tyr	Asp	Gln	Asn	Ser	Ala
CPV-2b (24)	Leu	Thr	Val	Ile	Ala	Gly	Tyr	Ser	Tyr	Asp	Gln	Asp	Ser	Ala
CPV-2c (8)	Leu	Thr	Ile	Ile	Ala	Gly	Tyr	Thr	Tyr	Asp	Gln	Glu	Thr	Ala

Ala=Alanine, Asn=Asparagine, Asp=Aspartic acid, Gln=Glutamine, Gly=Glycine, Ile=Isoleucine, Leu=Leucine, Lys=Lysine, Ser=Serine, Thr=Threonine, Tyr=Tyrosine, Val=Valine, CPV: Canine parvovirus

### Phylogenetic analysis

The phylogenetic tree shows an evolutionary separation between the CPV-2a, CPV-2b, and CPV-2c from Ecuadorian strains and the original CPV-2 used for vaccine production (C3). In this analysis, CPV-2a and CPV-2b were related to previous strains identified in Ecuador; however, the two strains of CPV-2a were clustered in different groups. Sample 4 clustered with a widely distributed variant of CPV-2a from around the world and sample 1 showed a more specific distribution in countries such as Argentina, Brazil, Africa, and India. Sample 8 seems to be related with a common ancestor for CPV-2b and CPV-2c strains widely distributed in Europe, Asia, and America (Figure-2). Test for recombination was performed with the Recombination Detection Program software v. 4.94, using the RDP, GENECONV, MaxChi, Bootscan, and SiScan programs, showing no evidence of recombination in the results.

## Discussion

CPV is a widely distributed virus that affects susceptible young dogs around the world. Molecular characterization is based on typing residue 426 inside the *VP2* gene without sequencing the complete (ORF) [27-29]. In our study, we characterized 35 samples of positive CPV collected from domestic puppies with clinical signs for CPV, and we analyzed the complete *VP2* gene of 4/35 samples randomly selected for each CPV variant (CPV-2a, CPV-2b, and CPV-2c). The isolated CPV strains belong to Quito, Ecuador, where the dog population has reached an estimated 300,000 animals, of which 40% are street dogs [30]. Therefore, the prevalence of CPV cannot be determined exactly. CPV-2a and CPV-2c showed the highest prevalence in our results (57.1% and 34.3%, respectively), demonstrating the spread of these variants in South America; in addition, previous studies revealed the presence of the same variants in Brazil, Argentina, Uruguay, and Ecuador, along with preliminary reports of CPV-2c in Colombia that were not yet confirmed [13-16,31]. According to the phylogenetic tree, two lineages of CPV-2a are present in Ecuador. One of them is grouped with lineages from North America, Europe, and Asia, and the second one, in addition to sharing a common ancestor with strains from Argentina, Brazil, India, and South Africa, seems to be exclusive to Ecuador, determining the possibility of viral circulation in a small geographical area of South America. Parvovirus infections are determined by an immature immunological system or a susceptible immune system in adult dogs [2]. Natural infections of CPV-2c produce similar signs of CPV-2a and CPV-2b, such as vomiting, diarrhea, fever, anorexia, depression, and leukopenia, despite some studies showing reduced mortality and a less-severe clinical course in non-vaccinated puppies of 9-10 weeks of age [11]. The new variants that emerged since 1970 with the almost complete worldwide replacement of CPV-2 with CPV-2a in 1980 [32] and the subsequent variants CPV-2b and CPV 2c that were rapidly spread between dog populations [7,8,33] became of special interest due to the new phenotypical characteristics, and the capability to adapt to host ranges different from wild and domestic dogs. Reports of severe clinical signs were obtained related to CPV-2c in domestic cats [34] and the capacity of the CPV-2a and CPV-2b variants to replicate in cats [35,36] including the possibility of coinfections between FPV and CPV-2a in young kittens [37]. Vaccines containing the original strain of CPV-2 seem to give protection against the variant CPV-2b, showing that it prevents an increase in acute phase proteins (α-1 acid glycoprotein and serum amyloid A), lymphopenia, neutropenia, and a reduction of the levels of neutrophil-CD4 expression when experimental dogs were inoculated orally and intranasally with a strain of CPV-2b after the application of three doses of a conventional modified live CPV-2 vaccine at 6, 9, and 12 weeks of age [38]. Evolutionary changes within the viral genome occurred since CPV-2 appeared in 1978 in the United States [39], producing the emergence of new variants of CPV-2, which involves structural aa substitutions in the capsid of the virus, also resulting in antigenic changes for each variant [40]. There were more than six AA changes in the capsid protein that differed from the new variants when compared with the original CPV-2 [32], but one specific mutation located in residue 426 of the *VP2* gene [9] resulted in a change of Asn-Asp-Glu between CPV-2a, CPV-2b, and CPV-2c, respectively, and this produced a biological change to be considered in natural infections within the dog population. A recent discovery of a new mutation in residue 514 (Ala-Ser) was reported in Argentina [41] and Colombia [16], and our findings showed a similar mutation in this residue. Therefore, we can consider the possibility of a new variant of CPV-2a emerging in South America that could possibly be related to a poor protection in vaccinated dogs (samples 1, 10, and 19), without discarding that the lack of information of vaccination schedule in these animals make difficult to support entirely this hypothesis. We can also mention that the biological impact of this single substitution (Ala514Ser) is not determined, making necessary to perform more studies to define the real impact of this change in the *VP2* protein. A substitution in residue 139 of Val, replacing Ile in sample 8 (CPV-2c), was also shown in isolates from Hungary belonging to CPV-2 strains [42]. An unusual mutation in residue 297 from Ser to Asn was present in sample 1 (CPV-2a), which was not being reported consistently in parvovirus research, with just one strain isolated in Argentina [43]. The mutation present in sample 24 (CPV-2b) in residue 322 from Thr to Ser was also a change reported only in China (2012) and South Korea (2007) (accession codes: KJ438802 and FJ197823). The mutation in residue 324 of sample 1 (CPV-2a) is a widely distributed change that was reported in Uruguay, China, Taiwan, and Nigeria [31,44-46]. Our results showed a prevalence of 20/35 (57.1%) for CPV-2a, 3/35 (8.5%) for CPV-2b, and 12/35 (34.3%) for CPV-2c. These variants were genetically characterized and demonstrated the complete spread of every known CPV-2 variant in Quito City, and our results corroborated the results of Aldaz *et al*. [15], who described an important prevalence of CPV-2a and CPV-2c in the central region of Ecuador. We also confirmed the spread of CPV-2c in South America [12-15]. Ribbon diagrams detailed in Figure-3 show substitutions along the structure of *VP2* protein. The EC/C3 (CPV-2) was used to construct the 3D view of the *VP2* protein and the location of all substitutions of the four complete sequences is located in each segment. Among the aa substitutions of sample 1 in residues within Loop 1-4 already reported by other researchers [7,33], the additional substitution in residue 514 appears out of the most antigenic region of the protein [47], not making clear the possible effect that this may have on the antigenic function of the viral capsid. Substitution in residue 514 is shown in Figure-4, showing no more structural changes than a loss of a molecule of oxygen.

## Conclusion

Considering these results, we can conclude that the presence of these variants in Ecuador corresponds with those described and distributed around the world, and the isolates that show the new mutations should be studied as possible new variants or subvariants emerging in this region of South America.

## Authors’ Contributions

DD: Wrote the draft paper and executed part of experiments; EM, BP, and LE collected, prepared the samples, and revised the manuscript; CA contributed to the design of the experiments and revised the manuscript, and APF designed, advised the experiments, and revised the manuscript. All authors read and approved the final manuscript.
